# Effect of the Addition of Diblock Copolymer Nanoparticles on the Evaporation Kinetics and Final Particle Morphology for Drying Aqueous Aerosol Droplets

**DOI:** 10.1021/acs.langmuir.3c02930

**Published:** 2023-12-21

**Authors:** Barnaby E.A. Miles, Derek H.H. Chan, Spyridon Varlas, Lukesh K. Mahato, Justice Archer, Rachael E.H. Miles, Steven P. Armes, Jonathan P. Reid

**Affiliations:** † School of Chemistry, University of Bristol, Bristol BS8 1TS, U.K.; ‡ Dainton Building, Department of Chemistry, University of Sheffield, Brook Hill, Sheffield S3 7HF, South Yorkshire, U.K.

## Abstract

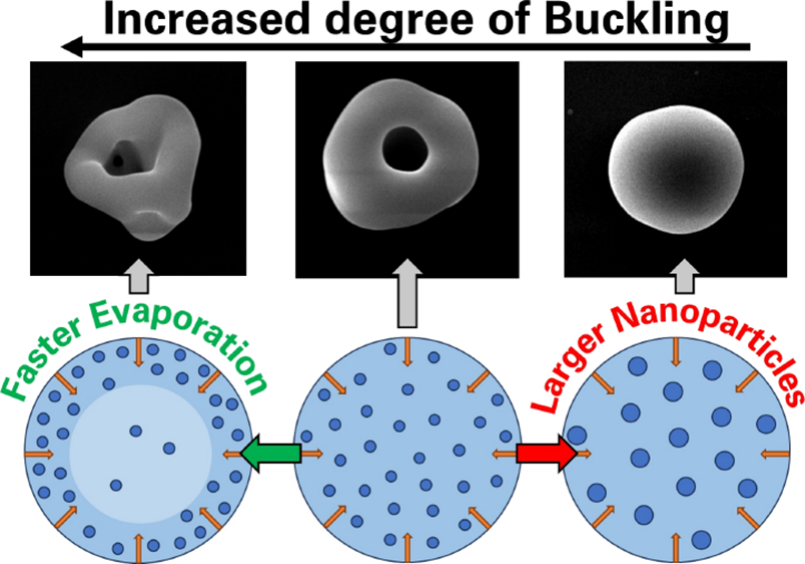

A deeper understanding of the key processes that determine the particle morphologies generated during aerosol droplet drying is highly desirable for spray-drying of powdered pharmaceuticals and foods, predicting the properties of atmospheric particles, and monitoring disease transmission. Particle morphologies are affected by the drying kinetics of the evaporating droplets, which are in turn influenced by the composition of the initial droplet as well as the drying conditions. Herein, we use polymerization-induced self-assembly (PISA) to prepare three types of sterically stabilized diblock copolymer nanoparticles comprising the same steric stabilizer block and differing core blocks with *z*-average diameters ranging from 32 to 238 nm. These well-defined nanoparticles enable a systematic investigation of the effect of the nanoparticle size and composition on the drying kinetics of aqueous aerosol droplets (20–28 μm radius) and the final morphology of the resulting microparticles. A comparative kinetics electrodynamic balance was used to obtain evaporation profiles for 10 examples of nanoparticles at a relative humidity (RH) of 0, 45, or 65%. Nanoparticles comprising the same core block with mean diameters of 32, 79, and 214 nm were used to produce microparticles, which were dried under different RH conditions in a falling droplet column. Scanning electron microscopy was used to examine how the drying kinetics influenced the final microparticle morphology. For dilute droplets, the chemical composition of the nanoparticles had no effect on the evaporation rate. However, employing smaller nanoparticles led to the formation of dried microparticles with a greater degree of buckling.

## 1 Introduction

Aerosols are multiphase systems consisting of a dispersed condensed phase (as discrete solid particles or liquid droplets, 100 nm to 100 μm diameter) within a gas phase. In many cases, condensed phase droplets can be assumed to be homogeneous in terms of their composition. This simplification is often made when treating the phase, volatility, reactivity, and optical properties of aerosols in consumer and agrochemical sprays, inhalable drug formulations, and atmospheric particulate matter. However, heterogeneities often arise owing to (i) surface enrichment in rapidly drying systems (e.g., in spray dryers),^1,2^ (ii) liquid–liquid phase separation (e.g., between inorganic and organic components within atmospheric aerosols),^3,4^ and (iii) limited mobility within viscous phases (e.g., in the case of exhaled viscous respiratory aerosol).^5,6^ Indeed, the condensed phase may also contain dispersed insoluble particles (e.g., nanoparticles, bacteria, liposomes, and emulsions) that vary in size from nanometers to micrometers;^7^ we refer to the latter as included particles (i.e., nanoparticles dispersed within a host droplet). Such complex aerosols have received very little attention, particularly with regard to the complex interplay of dynamic changes in the composition and size of the host droplets (e.g., during evaporation or condensation) with the interactions between them and the transport of included particles. Understanding this interplay is critical for predicting the final morphology of engineered microparticles (e.g., for spray-dried pharmaceutics), their light scattering and absorption cross-sections (e.g., for atmospheric optics), and the infectivity of respiratory pathogens (e.g., in exhaled aerosols).

The drying kinetics of solvent–solute aerosols leading to solidification/crystallization has been studied extensively.^2^ However, the complex phase behavior and structural evolution of drying aerosols containing included particles, which is analogous to the well-known “coffee ring” effect observed during drying of multiphase droplets on a surface, has received comparatively little attention.^7−11^ During evaporation, enrichment of the droplet surface by adsorption of the included particles can be inferred when the dimensionless Péclet number (*Pe*) ≫ 1. *Pe* is defined as the ratio of the time-dependent rate of retraction of the evaporating droplet surface (κ(*t*), m^2^ s^–1^) to the rate of diffusional mixing of solutes or included particles, which is dependent on the time-dependent diffusion constant, *D*(*t*) (m^2^ s^–1^).^8,12−15^ While κ(*t*) can be inferred from size measurements of the evaporating droplet, estimates of *D*(*t*) rely on models that capture the evolving composition and droplet viscosity. For aerosol droplets containing both soluble and insoluble components, it has been established that drying with different *Pe* values results in differing dried particle morphologies. For example, fast-drying aqueous NaCl droplets have a high *Pe* and produce particles with a framboidal (raspberry-like) morphology.^16^ Rapid surface enrichment results in multiple nucleation sites, which form multiple small crystals that undergo coalescence. At lower evaporation rates (smaller *Pe*), each droplet remains well-mixed as it dries, resulting in the formation of single crystal particles.^16,17^

**Figure 1:**
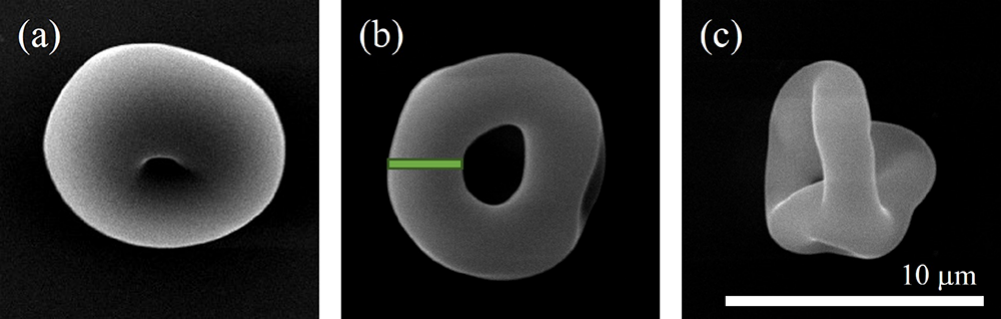
Representative SEM images illustrating dried microparticles with various morphologies: (a) crinkled/dimpled, (b) donut, and (c) burst. The annular width is defined as the mean outer radius of a particle minus its mean cavity radius and is shown by the green band in b.

Droplets with insoluble inclusions form a surface layer of densely packed nanoparticles as they dry; this initial layer is denoted the nanoparticle shell.^18^ The *Pe* of the drying droplet influences the radius and thickness of this shell.^8^ A larger *Pe* results in a higher degree of surface enrichment at an earlier stage during the drying process, leading to a radially larger but thinner shell. By comparison, a lower *Pe* results in droplets drying to form smaller but thicker shells; for such droplets, the slower evaporation rate allows the droplet to remain well mixed until a lower droplet surface area is attained. If the *Pe* value is sufficiently low, then a dense sphere is formed.^18,19^ Once the shell is formed, water continues to evaporate from within the encased structure, lowering the pressure within the shell and increasing the capillary pressure between the droplet surface and the gas phase. This higher capillary pressure builds compressive stress in the shell. If the compressive stress exceeds a certain critical value, then the shell undergoes buckling to form crinkled, doughnut, or burst morphologies (Figure 1).^20−22^ The critical pressure within a particle shell depends on the shell radius and thickness as well as the size of the included nanoparticles. The threshold for buckling can be represented as the ratio between the nanoparticle size and the shell radius^23^
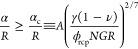
1where α is the nanoparticle radius, *R* is the radius of the droplet at which the particle shell first forms, *A* is a numerical constant, γ is the surface tension of the droplet, ν is the Poisson ratio, ϕ_rcp_ is the random close-packing volume fraction, *N* is the number of contacting neighbors, and *G* is the shear modulus.

Previous investigations of drying aqueous aerosol droplets with insoluble inclusions have focused on hard nanoparticles such as silica and polystyrene.^8,18,20,24^ The impact of the droplet composition and drying conditions (e.g., temperature and relative humidity (RH)) on the *Pe* number and final microparticle morphology was examined for dilute (0.10–0.60% *v*/*v*) aqueous silica aerosols by Archer et al. (2020).^3^^,^^8^ For this system, either lowering the RH or raising the temperature increased the *Pe* value within the drying droplets, resulting in a higher degree of buckling in the final microparticles. The influence of the initial droplet size on surface enrichment and the resulting morphology of spray-dried silica nanoparticles (2% *w*/*w*) was examined by Bahadur et al.^9,18^ Larger droplets exhibited less surface enrichment and therefore produced larger, less dense microparticles. The critical buckling conditions in the case of silica and polystyrene latex particles have been identified by Bamboriya and Tirumkudulu.^11,20^ The hardness and the size of such included nanoparticles governed the resultant particle morphology for sessile-dried droplets.

There have been very few prior studies of the relationship between the drying kinetics of individual nanoparticle-laden droplets and the physical properties of the final dried microparticles. Unlike the drying of relatively large sessile droplets (i.e., for droplet volumes ranging from microliters to nanoliters) on surfaces, monitoring the drying of transient droplets of sub-nanoliter dimensions within short timescales (within 5 s) is a formidable technical challenge. It is also difficult to obtain reproducible measurements for droplets of the same size and composition to investigate the impact of dynamic processes on the morphology of the final dried microparticles. Previously, Ivey et al.^25^ investigated the effect of varying the temperature, solvent type, and droplet size on the physical properties of dried microparticles obtained after drying monodisperse droplets within a spray dryer. Archer et al. (2020)^8^ studied the effect of the temperature and RH on the drying kinetics of silica nanoparticle-loaded droplets and how such parameters affected the morphology of the final dry microparticles. However, the present study is the first to investigate and experimentally validate the link between the drying kinetics, nanoparticle diameter, and dry microparticle morphology obtained from freely drying nanoparticle-laden droplets. Furthermore, the excellent control over the drying conditions, reproducibility for the drying event, uniform initial droplet diameter, and near-monodisperse nanoparticles leads to highly reproducible morphologies for the final dried microparticles. Such data provide a timely opportunity to develop a quantitative framework to predict and control specific microparticle morphologies, as is commonly achieved for sessile droplet drying.^11,20^

**Figure 2:**
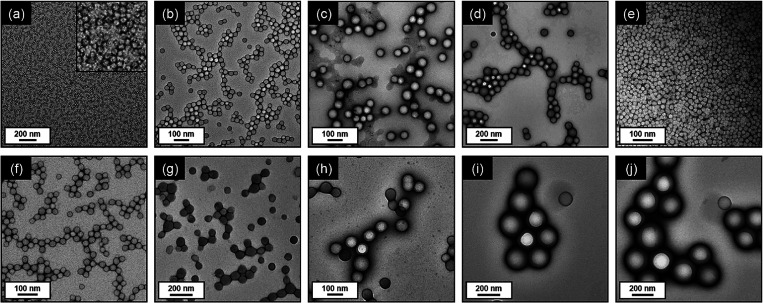
Representative TEM images recorded for the diblock copolymer nanoparticles prepared by PISA. (a) PGMA_50_-PBzMA_50_. (b) PGMA_50_-PBzMA_100_. (c) PGMA_50_-PBzMA_200_. (d) PGMA_50_-PBzMA_300_. (e) PGMA_50_-PMMA_100_. (f) PGMA_50_-PTFEMA_100_. (g) PGMA_50_-PTFEMA_200_. (h) PGMA_50_-PTFEMA_300_. (i) PGMA_65_-PBzMA_1000_. (j) PGMA_50_-PBzMA_1000_.

In this study, we chose to examine three types of diblock copolymer nanoparticles: poly(glycerol monomethacrylate)–poly(benzyl methacrylate) (PGMA_*x*_-PBzMA_*y*_), poly(glycerol monomethacrylate)–poly(methyl methacrylate) (PGMA_*x*_-PMMA_*y*_), and poly(glycerol monomethacrylate)–poly(trifluoroethyl methacrylate) (PGMA_*x*_-PTFEMA_*y*_), where *x* and *y* denote the average number of monomer repeat units per block. PGMA was selected as a convenient non-ionic steric stabilizer block, which has been extensively studied by Armes and Warren.^26^ The nanoparticles are prepared directly in aqueous media using polymerization-induced self-assembly (PISA).^26^ This approach produces sterically stabilized nanoparticles of readily tunable sizes (see Figure 2) in which the nanoparticle cores are composed of hydrophobic PBzMA, PMMA, or PTFEMA chains, while the PGMA chains confer steric stabilization.^27−29^ It is the variety in the chain lengths that allowed for a systematic variation of the particle size while maintaining relatively narrow particle size distributions.^26,28^ The glass transition temperatures of PBzMA and PTFEMA are 54 and 55 °C, respectively.^30,31^ These values are significantly lower than that of polystyrene (100 °C) and silica (1207 °C).^15,32^ Hence, such nanoparticles are rather softer (exhibit a lower compressive stress) at 20 °C than those reported in prior studies. The literature densities of the three nanoparticle cores are 1.18 g cm^–3^ for PBzMA, 1.19 g cm^–3^ for PMMA, and 1.47 g cm^–3^ for PTFEMA.^27,33,34^ These sterically stabilized nanoparticles possess a neutral character unlike the charge-stabilized anionic silica nanoparticles recently reported by Archer et al. (2020).^8^

## 2 Experimental Section

### 2.1 Synthesis of Diblock Copolymer Nanoparticles

#### 2.1.1 Materials

Methyl methacrylate (MMA; 99%), 2,2,2-trifluoroethyl methacrylate (TFEMA; 99%), benzyl methacrylate (BzMA; 98%), 4,4’-azobis(4-cyanopentanoic acid (ACVA; 98%), and 2-cyano-2-propyl benzodithioate (CPDB; 97%) were purchased from Sigma-Aldrich (UK). Glycerol monomethacrylate (GMA; 99.8%) was kindly donated by GEO Specialty Chemicals (Hythe, UK). Deionized water obtained from an Elga Medica DV25 water purification unit was used for all experiments.

### 2.2 Synthesis of the PGMA Precursor

A typical protocol for the synthesis of a PGMA precursor was conducted as follows. The GMA monomer (30.0 g, 187 mmol), CPDB (0.589 g, 2.66 mmol; target PGMA DP = 70), ACVA (0.149 g, 0.53 mmol; CPDB/ACVA molar ratio = 5.0), and ethanol (46.5 g, 60% *w*/*w*) were weighed into a 250 mL round-bottom flask. This flask was immersed in an ice bath, and the solution was degassed with N_2_ gas for 30 min. The flask was then placed in an oil bath set at 70 °C for 165 min before quenching the GMA polymerization by exposing the reaction mixture to air while cooling to 20 °C. A GMA monomer conversion of 71% was determined by ^1^H NMR spectroscopy. Methanol (30 mL) was added to the flask, and the crude homopolymer was precipitated into a 10-fold excess of dichloromethane (three times). A mean degree of polymerization (DP) of 50 was determined by end-group analysis via ^1^H NMR spectroscopy (the integrated aromatic proton signals at 7.4–7.8 ppm were compared to the methacrylic backbone protons at 0.7–2.5 ppm). A similar protocol was employed to prepare the PGMA_65_ precursor by weighing the following reagents into a round-bottom flask: GMA monomer (10.0 g, 62.4 mmol), CPDB (0.173 g, 0.780 mmol; target PGMA DP = 80), ACVA (0.044 g, 0.156 mmol; CPBD/ACVA molar ratio = 5.0), and ethanol (15.3 g, 60% *w*/*w*). In this case, the final GMA conversion was 81% after 165 min at 70 °C.

### 2.3 Synthesis of Diblock Copolymer Nanoparticles via RAFT Aqueous Emulsion Polymerization

A typical protocol for the synthesis of PGMA_50_-PBzMA_50_ diblock copolymer nanoparticles was conducted as follows. The PGMA_50_ precursor (0.100 g, 12.2 μmol), BzMA monomer (0.107 g, 0.61 mmol, target DP = 50), ACVA initiator (0.680 mg, 2.43 μmol, PGMA_50_/ACVA molar ratio = 5.0), and deionized water (1.871 g, 10% *w*/*w* solution) were weighed into a 10 mL round-bottom flask, and the solution pH was adjusted to pH 7 using 1 M NaOH. The reaction mixture was cooled with the aid of an ice bath and degassed with N_2_ gas for 30 min. The flask was placed in an oil bath set at 70 °C. After 6 h, the flask was removed and its contents were exposed to air to quench the polymerization while cooling to 20 °C. A final BzMA conversion of 99% was indicated by ^1^H NMR spectroscopy (the vinyl signals for the residual monomer at 5.2–6.2 ppm were compared to the methacrylic backbone protons assigned to the copolymer at 0.7–2.2 ppm). The PGMA_50_-PBzMA_100–1000_ nanoparticles, PGMA_65_-PBzMA_1000_ nanoparticles, and PGMA_50_-PTFEMA_100–300_ nanoparticles were prepared using a similar protocol. For higher core-forming block DPs, the mass of the PGMA precursor and the ACVA initiator remained constant (PGMA/ACVA molar ratio = 5.0). The target DP was varied by increasing the mass of either the BzMA or TFEMA monomer while adjusting the mass of water to ensure that all syntheses were prepared at 10% *w*/*w* solids. PGMA_50_-PMMA_100_ nanoparticles were also prepared using the same protocol. However, in this case, the overall reaction time was 3 h at 70 °C. In all cases, ^1^H NMR spectroscopy studies confirmed more than 99% conversion of the BzMA, MMA, or TFEMA. Moreover, gel permeation chromatography (GPC) analyses (DMF eluent) indicated a high blocking efficiency (i.e., efficient chain extension) for the PGMA precursor, suggesting the formation of well-defined amphiphilic diblock copolymer chains (Figure S1). The hydrodynamic *z*-average diameter was determined from DLS studies of 0.1% *w*/*w* aqueous dispersions of such nanoparticles at 20 °C using a Malvern Zetasizer NanoZS instrument. Scattered light was detected at a fixed scattering angle of 173° with measurements averaged over three runs. Table 1 summarizes the nanoparticle composition, *z*-average diameter, and polydispersity index (PDI) obtained for each nanoparticle type. Transmission electron microscopy (TEM) images were obtained for dried dilute aqueous dispersions at an acceleration voltage of 100 kV using a Philips CM100 microscope equipped with a Gatan 1k CCD camera.

**Table 1:** Summary of the Chemical Composition and DLS Data Obtained for the 10 Examples of Diblock Copolymer Nanoparticles Examined in This Study

sample ID	diblock composition	DLS diameter (nm)	DLS PDI
1	PGMA_50_-PBzMA_50_	32	0.03
2	PGMA_50_-PBzMA_100_	36	0.04
3	PGMA_50_-PBzMA_200_	64	0.06
4	PGMA_50_-PBzMA_300_	79	0.04
5	PGMA_50_-PMMA_100_	35	0.08
6	PGMA_50_-PTFEMA_100_	31	0.04
7	PGMA_50_-PTFEMA_200_	40	0.08
8	PGMA_50_-PTFEMA_300_	64	0.07
9	PGMA_65_-PBzMA_1000_	214	0.04
10	PGMA_50_-PBzMA_1000_	238	0.06

### 2.4 Droplet Drying Measurements

The ability to confine single aerosol droplets through noncontact levitation to study their drying histories and phase behavior has been explored extensively in the literature.^35,36^ Here, we used a comparative kinetic electrodynamic balance (CK-EDB) capable of levitating single aerosol droplets within an RH and temperature-controlled environment by application of AC and DC voltages to a pair of vertically opposed, concentric cylindrical electrodes. A small charge was conferred on the generated droplet, which was held between the upper and lower electrode pairs by the strong electric field generated from the applied voltages. The CK-EDB has been documented extensively in our previous publications and will only be briefly discussed here.^2,8,37−40^ A schematic of the experimental setup can be found in Figure S2a.

Single aqueous aerosol droplets of a known charge polarity (approximately −30 fC) and initial radius (28 ± 3 μm), containing diblock copolymer nanoparticles of varying sizes (31–238 nm diameter) were dispensed by a droplet-on-demand generator (MicroFab, MJ-ABP-01, orifice = 30 μm diameter) into the stable trapping region of the CK-EDB.^2,8^ Each droplet was illuminated by a 532 nm laser (Laser Quantum, Ventus continuous wave [CW]), and the resulting near-forward elastically scattered laser light was collected with a CCD camera over an angular range of ∼24° centered at 45° to the propagation direction of the laser beam. A nitrogen gas flow of 200 mL min^–1^ at 20 °C and a set RH (ranging from ∼0% to > 90%) was passed through the inner (lower) electrode and directly over the droplet surface. Evaporation leads to a reduction in the droplet mass, so the position of the droplet at the center of the trap was maintained by adjusting the magnitude of the DC voltage applied to the lower concentric electrode. The temporal evolution of the droplet radius (i.e., its drying history) was obtained by comparing the angularly resolved scattered light intensity collected from the droplet (the phase function) with the geometrical optics approximation, making appropriate corrections for changes in the density and refractive index.^40,41^ During the initial stages of evaporation, the aqueous droplet remains spherical and homogeneous, producing regularly spaced interference fringes in the droplet phase function (see Figure S2b). However, the formation of a nanoparticle shell at the surface of the trapped droplet causes a rapid loss in its sphericity and its internal composition becomes inhomogeneous. This is illustrated by the phase function of the droplet shown in Figure S2c, where a clear change in the form occurs, which produces irregular fringes and indicates the point at which the particle shell forms.

### 2.5 Collection of Dried Microparticles

To complement the droplet evaporation data obtained using the CK-EDB, a falling droplet column (FDC) was used to dry multiple aqueous aerosol droplets containing the diblock copolymer nanoparticles and collect the resulting dried microparticles. The morphology of these microparticles was then characterized using scanning electron microscopy (SEM). A schematic representation of the FDC is shown in Figure S3. A full description of the experimental setup has been given previously^16^ so only a brief review is provided here. A droplet-on-demand dispenser (Microfab, MJ-ABP-01, orifice = 30 μm diameter) was used to generate a stream of uniform droplets of known composition, which is the same as the droplets used in the drying measurements. These were injected horizontally into a vertically aligned 50 cm long glass column with the droplet stream aligned such that the particles fell vertically down the center of the column upon the loss of the horizontal component of their velocity. The droplets dried as they fell, forming dry microparticles. A glass slide at the bottom of the column was used to collect the dried microparticles with a vertically propagating 532 nm wavelength laser serving as an alignment guide to ensure that the dry microparticles deposited onto the glass slide instead of striking the column walls.

The RH inside the glass column was controlled by combining humidified and dry nitrogen gas flows with the RH being determined by the flow ratio. The temperature inside the column was 20 °C across all experiments. Both the RH and temperature were continuously recorded using an RH probe and K-type thermocouple, respectively. The glass slides containing the dried microparticles were attached to an aluminum stub and sputter-coated with a thin overlayer of silver. The stub was placed in the SEM instrument (JSM-IT300, JEOL), and images were recorded under an ultrahigh vacuum at 15 kV.

### 2.6 Modeling Evaporation Profiles for Data Validation

Evaporation profiles of pure water droplets were modeled using the Single Aerosol Drying Kinetics and Trajectories (SADKAT) model previously developed by us and compared to the evaporation profiles obtained for the nanoparticle-laden aerosol droplets. SADKAT is a free-to-use and open-source program that enables the calculation of complete droplet trajectories and evaporation profiles. Since it has been discussed in detail in previous publications, this program is only briefly reviewed here.^17,42^

Evaporation and condensation processes for droplets containing one volatile component and one involatile component can be accurately modeled using SADKAT, which accounts for both the solute effect and surface curvature.^43^ SADKAT was designed to be applied to droplets in the continuum regime (droplet diameters > 1 μm) but includes correction factors to allow an accurate simulation of smaller droplets. However, it cannot account for evaporation-induced surface enrichment and all droplets are assumed to be thermally and compositionally homogeneous. When simulating the pure water droplet evaporation profiles, all the input parameters required by SADKAT (RH, initial droplet diameter, ambient temperature, droplet temperature, and airflow rate) were obtained from experimental measurements.

## 3 Results and Discussion

### 3.1 Effect of the Nanoparticle Size and Core Composition on the Droplet Drying Kinetics

For aqueous aerosol droplets containing charge-stabilized silica nanoparticles, the nanoparticles simply act as a spectator and the evaporation rate of the droplet is independent of the initial composition (up to 0.50% *v*/*v* silica nanoparticles).^8^ To investigate whether similar behavior is observed for the softer sterically stabilized nanoparticles examined herein, evaporation rates were determined for (i) aqueous aerosol droplets containing PGMA-PBzMA nanoparticles of varying sizes and (ii) nanoparticles of comparable sizes but different core compositions (e.g., PGMA_50_-PMMA_100_ and PGMA_50_-PTFEMA_100_). This enabled a matrix of different nanoparticle sizes and compositions to be investigated.

**Figure 3:**
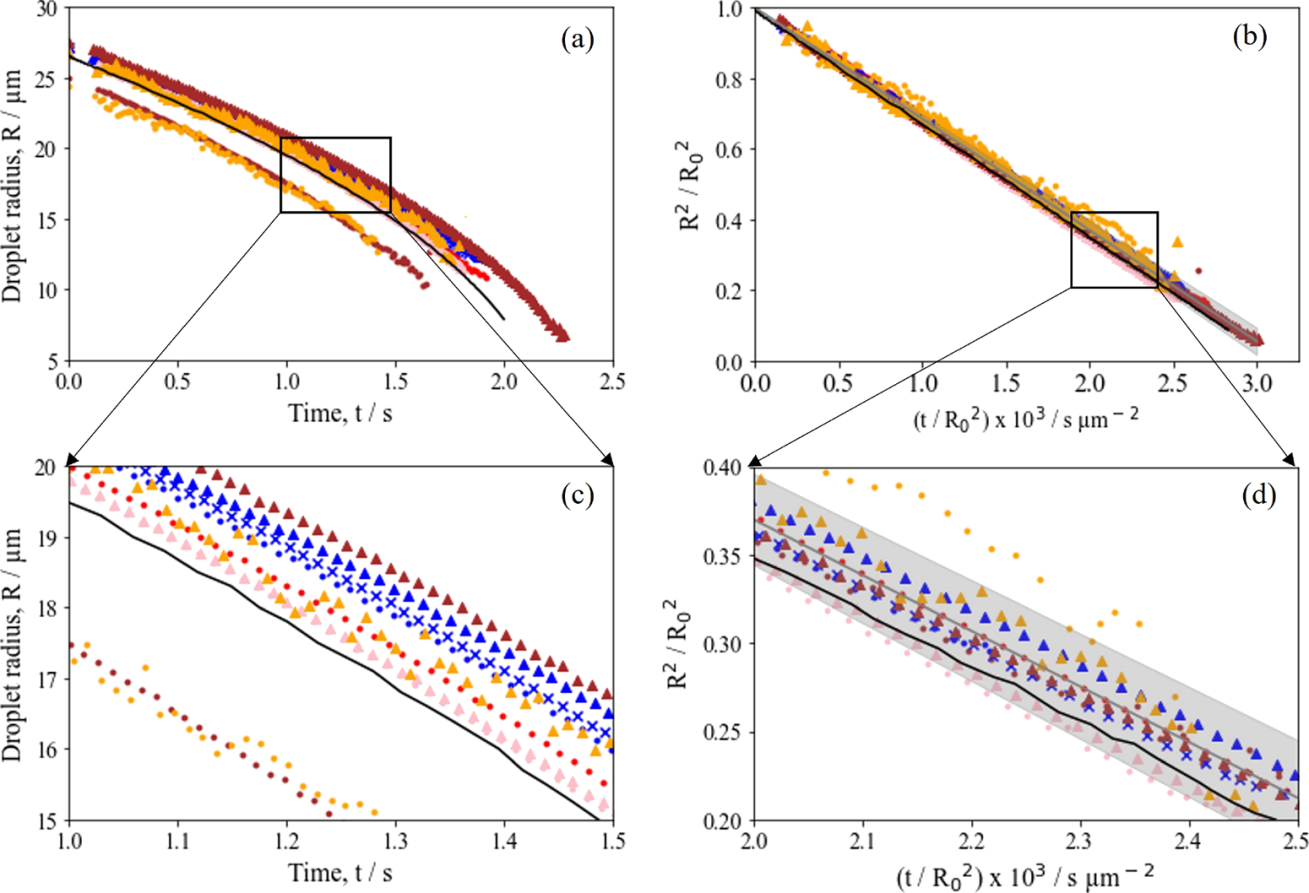
(a) Evaporation profiles obtained for all aqueous aerosol droplets (2% *w*/*w*) at RH = 0% and 293 K, compared with an evaporation profile for pure water using the SADKAT model. (b) Normalized radius squared plots for the data shown in a. (c) Expanded region of the box shown in a. (d) Expanded region of the box shown in b. The mean evaporation rate across all aqueous aerosol droplets is 315.0 ± 13.0 μm^2^ s^–1^. PGMA_50_-PBzMA_50_ is shown as red circles, PGMA_50_-PBzMA_100_ as blue circles, PGMA_50_-PBzMA_200_ as pink circles, PGMA_50_-PBzMA_300_ as brown circles, PGMA_50_-PMMA_100_ as blue crosses, PGMA_50_-PTFEMA_100_ as blue triangles, PGMA_50_-PTFEMA_200_ as pink triangles, PGMA_50_-PTFEMA_300_ as brown triangles, PGMA_65_-PBzMA_1000_ as orange circles, and PGMA_50_-PBzMA_1000_ as orange triangles. The evaporation rate of pure water is shown in black, and the average aqueous droplet evaporation rate is shown in gray.

Figure 3a shows the evaporation profiles (radius vs time) obtained for aqueous droplets containing the 10 types of nanoparticles summarized in Table 1. In each case, aerosols were prepared using a 2% *w*/*w* aqueous dispersion of nanoparticles and evaporated into a dry atmosphere (0% RH) at 293 K. Figure 3b shows the droplet evaporation profiles plotted in terms of the square of the normalized radius with the gradient of the resulting line giving the droplet evaporation rate. This follows from the “Radius-Square Law” indicated by eq 2(44):
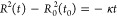
2Here, *R*^*2*^(*t*) is the square of the droplet radius at any given time, *R*_0_^2^(*t*_0_) is the square of the initial droplet radius, κ is the droplet evaporation rate, and *t* denotes the time.^8^

**Figure 4:**
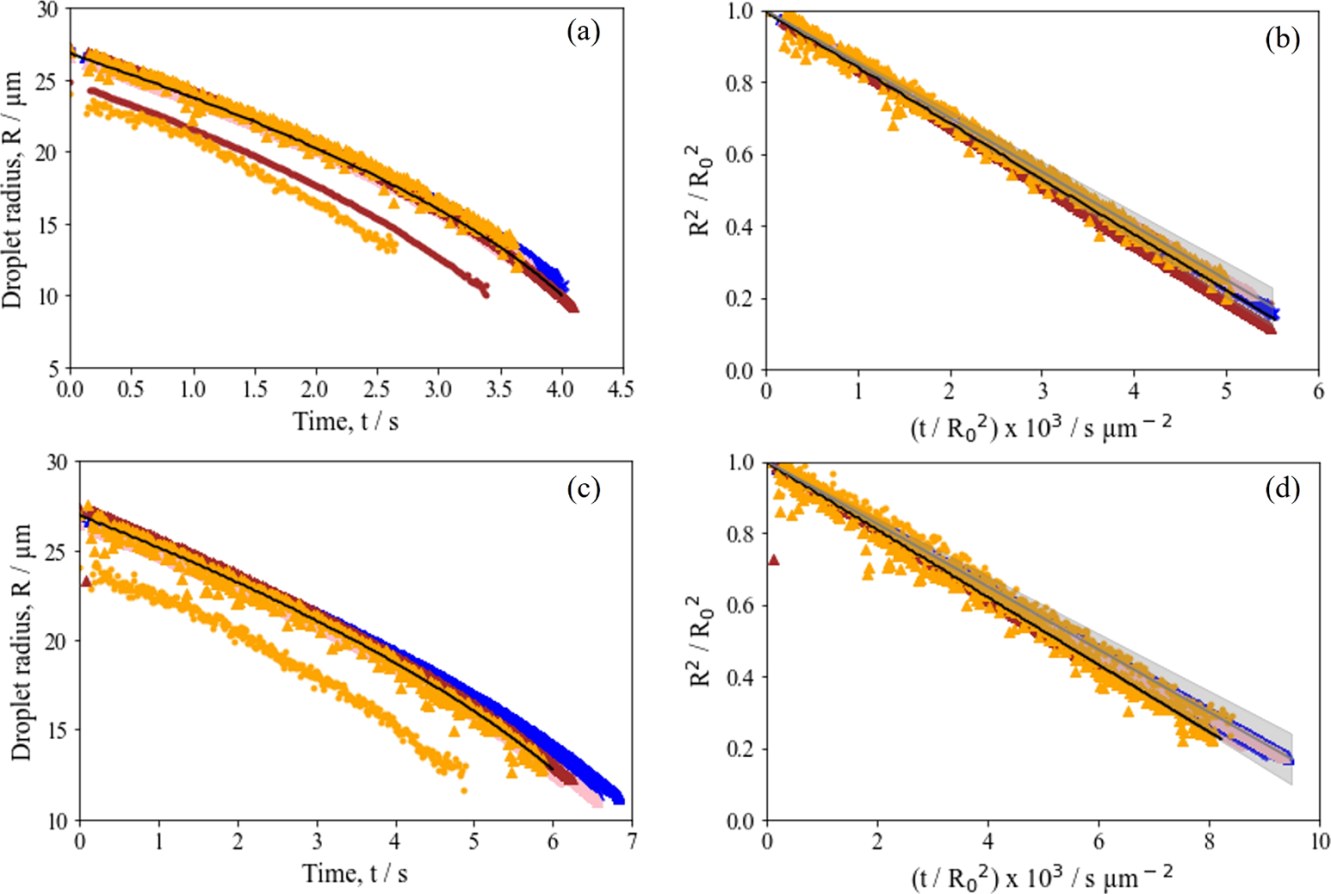
Evaporation profiles obtained for all aqueous aerosol droplets (2% *w*/*w*) compared with an evaporation profile modeled for pure water. Panels a and b show data for RH = 45% and *T* = 293 K with an average evaporation rate of 150.0 ± 9.8 μm^2^ s^–1^. Panels c and d show data for RH = 65% and *T* = 293 K with an average evaporation rate of 87.9 ± 7.5 μm^2^ s^–1^. PGMA_50_-PBzMA_50_ is shown as red circles, PGMA_50_-PBzMA_100_ as blue circles, PGMA_50_-PBzMA_200_ as pink circles, PGMA_50_-PBzMA_300_ as brown circles, PGMA_50_-PMMA_100_ as blue crosses, PGMA_50_-PTFEMA_100_ as blue triangles, PGMA_50_-PTFEMA_200_ as pink triangles, PGMA_50_-PTFEMA_300_ as brown triangles, PGMA_65_-PBzMA_1000_ as orange circles, and PGMA_50_-PBzMA_1000_ as orange triangles. The evaporation rate of pure water is shown in black, and the average aqueous droplet evaporation rate is shown in gray.

Given the similarity in the evaporation rates observed in Figure 3b, the rate of reduction in the droplet radius over time is clearly independent of the size and chemical composition of the nanoparticles within the droplet. Figure 4a,b also shows the evaporation rate of a pure water droplet under the same environmental conditions for comparison as modeled using SADKAT (κ = 321.3 μm^2^ s^–1^). This clearly lies within the uncertainty bounds of the average aerosol droplet evaporation rates. Thus, the diblock copolymer nanoparticles simply act as spectators during evaporation with the droplet evaporation rate remaining independent of the initial droplet composition (nanoparticle size and chemical composition) when employed at 2% *w*/*w*.

For PGMA_50_-PBzMA_1000_ and PGMA_65_-PBzMA_1000_ (samples 9 and 10, respectively), the inferred radius as a function of time was more erratic than that observed for the other types of nanoparticles. Such nanoparticles are the largest examined in the present study with mean diameters of 214 and 238 nm, respectively (see Table 1). Indeed, the corresponding experimentally observed droplet phase functions exhibited irregular interference fringe intensities.^40^ These two nanoparticles were sufficiently large that, as their concentration in the droplet increased through water evaporation, the inhomogeneity affected the light scattering. Consequently, the estimation of the mean droplet radius from the phase function was of reduced accuracy.

### 3.2 Effect of the Relative Humidity on the Droplet Drying Kinetics

In Section 3.1, we have shown that under suitably dry conditions (0% RH and 293 K), the nanoparticles within the aerosol droplets (2% *w*/*w*) merely act as spectators during evaporation. To investigate whether similar behavior is also observed under humid conditions, evaporation profiles for all nanoparticle-laden droplets at 45% RH and 65% RH were recorded using 2% *w*/*w* nanoparticle dispersions at 293 K. The results are shown in Figure 4. Evaporation rates obtained for all nanoparticle-laden droplets were comparable regardless of the RH value as judged by the similar normalized radius-squared dependencies shown in Figure 4b,d (45% RH and 65% RH, respectively). These measurements were also consistent with the predicted rate of evaporation for a pure water droplet drying under the same conditions as calculated using SADKAT (κ = 155.8 μm^2^ s^–1^ at 45% RH and κ = 94.4 μm^2^ s^–1^ at 65% RH). Thus, the evaporation rates of the aqueous aerosol droplets agree with the pure water droplet within the experimental error. This indicates that these sterically stabilized nanoparticles act as spectators independent of either the aerosol droplet drying that occurs under dry or humid conditions.

However, there is a discernible reduction in the aerosol droplet evaporation rate with an increasing RH regardless of the nanoparticle type, which is consistent with other studies.^2,8,16^

### 3.3 Impact of the Drying Kinetics on the Resultant Dried Particle Morphology

**Figure 5:**
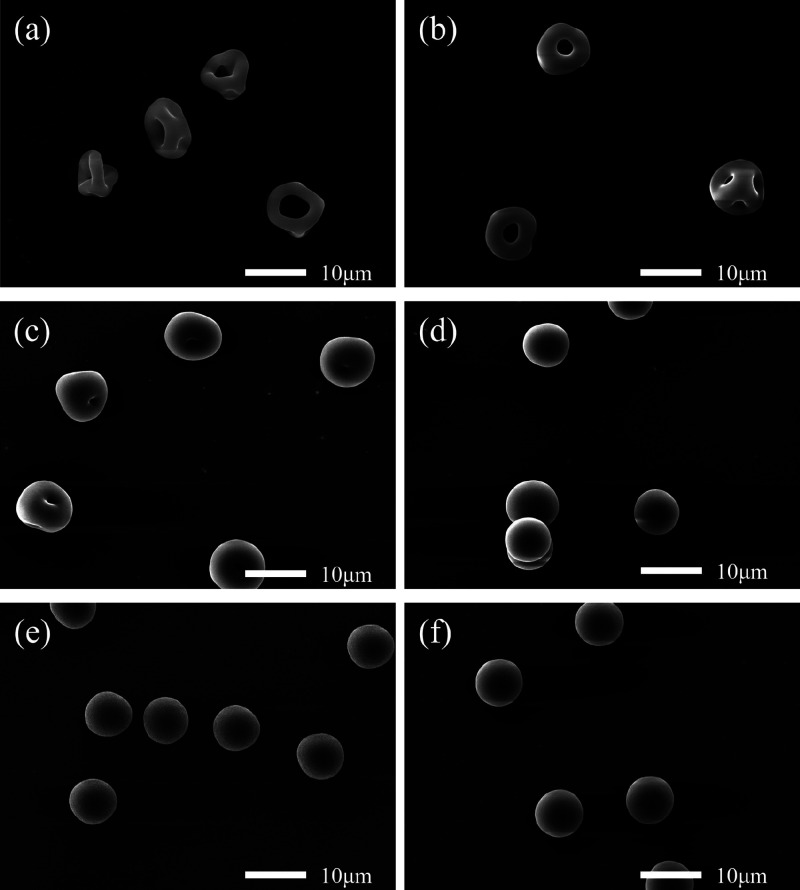
SEM images recorded for dried microparticles obtained after drying aqueous aerosol droplets containing 2% *w*/*w* PGMA_*x*_-PBzMA_*y*_ nanoparticles at 293 K; 32 nm-diameter PGMA_50_-PBzMA_50_ nanoparticles dried at (a) 0% RH and (b) 45% RH, 79 nm-diameter PGMA_50_-PBzMA_300_ nanoparticles dried at (c) 0% RH and (d) 45% RH, and 214 nm PGMA_65_-PBzMA_1000_ nanoparticles dried at (e) 0% RH and (f) 45% RH.

To investigate the effect of varying the evaporation rate on the final morphology, SEM images were recorded for microparticles produced at 293 K when drying aqueous aerosol droplets containing three types of nanoparticles [PGMA_50_-PBzMA_50_ (32 nm), PGMA_50_-PBzMA_300_ (79 nm), and PGMA_65_-PBzMA_1000_ (214 nm)]. Microparticles were collected after drying at 0% RH and 45% RH (Figure 5). PGMA_65_-PBzMA_1000_ nanoparticles were selected instead of PGMA_50_-PBzMA_1000_ nanoparticles simply because it proved difficult to generate uniform droplets when using the latter nanoparticles. Having a broad range of droplet sizes is problematic when collecting samples in the FDC as this affects the size of the final dry microparticles.

**Figure 6:**
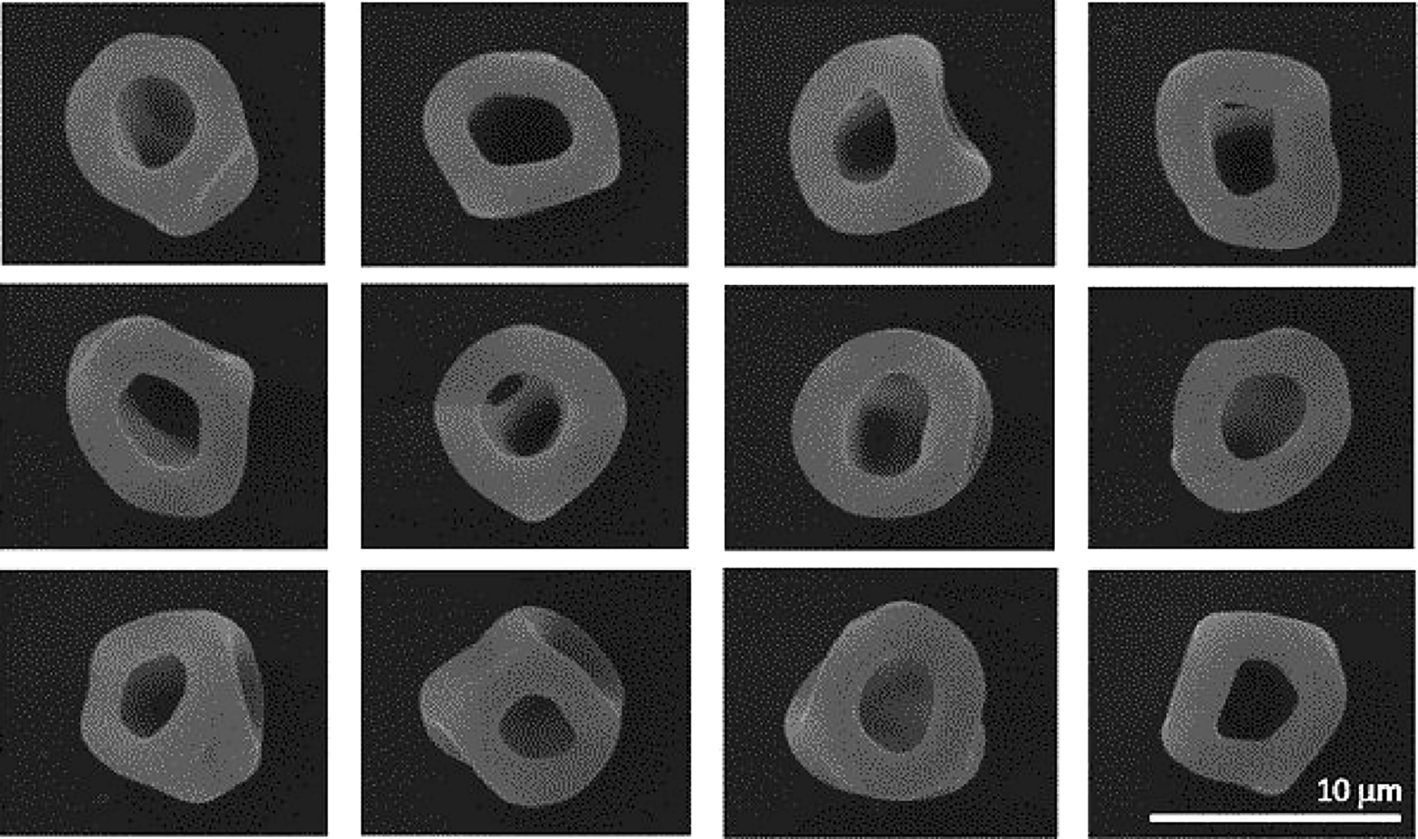
Library of SEM images recorded for microparticles obtained after drying aqueous aerosol droplets containing 2% *w*/*w* PGMA_50_-PBzMA_50_ nanoparticles at 0% RH and 293 K. Mean annular widths were estimated from the analysis of these 12 microparticles.

The annular width (i.e., the radius of the particle minus the radius of the cavity in the particle) of the final dry microparticles prepared using the 32 nm nanoparticles increased from 3.5 ± 0.2 μm (0% RH; Figure 5a) to 4.0 ± 0.1 μm (45% RH; Figure 5b), as estimated from the corresponding SEM images shown in Figure 6 and Figure S4a. Thus, dry conditions (i.e., faster droplet evaporation rates) lead to greater deformation of the microparticle morphology. Furthermore, 4 of the 13 analyzed microparticles exhibited a “broken donut” morphology at 0% RH with the remaining microparticles possessing a donut morphology. In contrast, all five microparticles exhibited a donut morphology when dried at 45% RH. Even when comparing the donut-like morphologies at each RH value, the microparticles generated at 0% RH are significantly more deformed, indicating a greater degree of buckling. The term “donut-like” is used here because the most common orientation of these microparticles displays a donut-like face pointing upward. However, inspecting Figure 5b and Figure S4a confirms that all microparticles actually contain two additional holes when viewed from an alternative perspective. We emphasize that the term “donut-like” does not imply that these dried microparticles contain only one hole.

**Figure 7:**
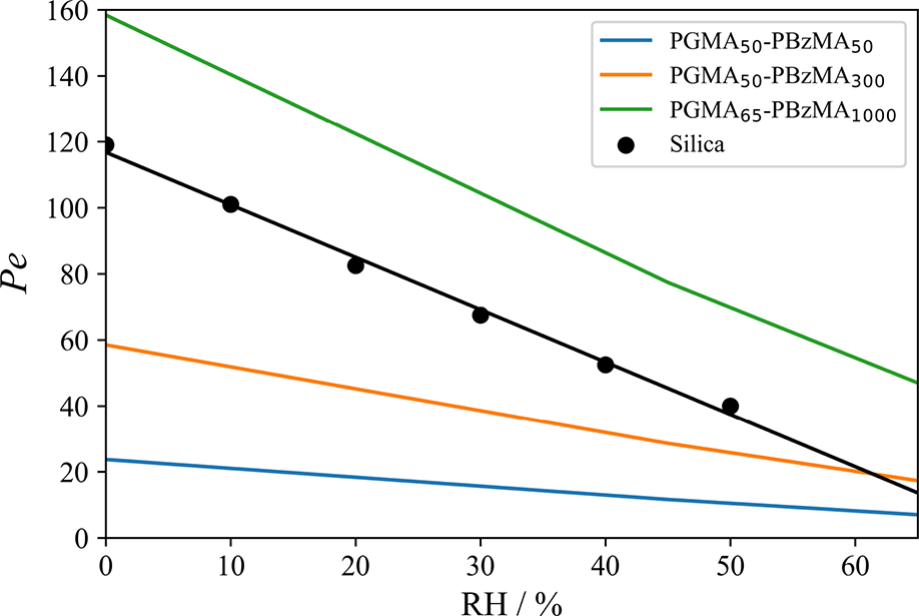
Calculated Peclet numbers for three examples of PGMA_*x*_-PBzMA_*y*_ nanoparticles as a function of RH. All Peclet numbers were calculated for droplets drying at 293 K. The Peclet numbers of aqueous aerosol droplets containing silica nanoparticles (180 nm, 0.40% *v*/*v*) at 293 K and various RH values from Archer et al. are included for comparison.^8^

Aqueous aerosol droplets containing 79 nm nanoparticles produced a dimpled morphology when dried at 0% RH but dense spheres at 45% RH. This indicates that buckling does not occur at a sufficiently slow evaporation rate (higher RH). This is consistent with the established relationship between RH and *Pe* for aqueous droplets and similar morphological observations have been reported for aqueous aerosol droplets containing silica nanoparticles.^8,20^ Increasing the droplet evaporation rate by lowering the RH from 45% to 0% leads to greater surface enrichment and hence a higher *Pe* (Figure 7). A higher *Pe* results in a larger microparticle shell radius, and therefore a smaller *a/R* value for a given nanoparticle diameter (eq 1). Microparticles undergo buckling below a critical *a*/*R* value, so reducing the *a*/*R* ratio within a drying aqueous aerosol droplet increases both the probability and the degree of buckling.

When drying aqueous aerosol droplets containing the 214 nm nanoparticles, the resulting microparticles formed dense spheres regardless of the humidity with an approximately constant particle diameter (7.6 ± 0.1 μm to 7.8 ± 0.1 μm (see Figure S4). Given that no buckling occurred when such aqueous droplets are dried at a relatively fast evaporation rate, then no change in the morphology should occur at lower evaporation rates.

### 3.4 Effect of the Nanoparticle Size on the Resultant Dried Particle Morphology

**Figure 8:**
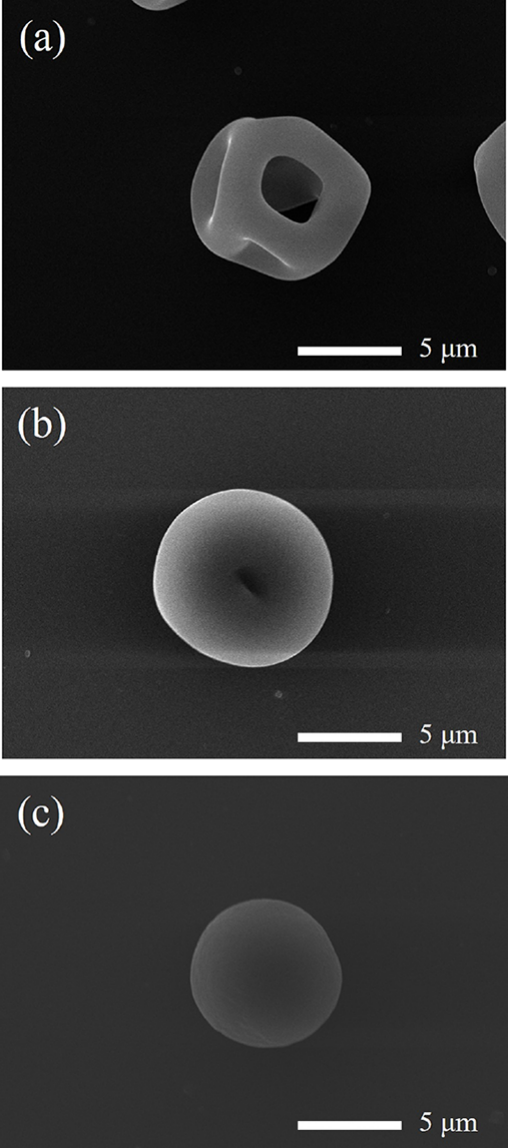
SEM images recorded for the final microparticles obtained when drying aqueous aerosol droplets containing 2% *w*/*w* nanoparticles at 0% RH and 293 K: (a) 32 nm-diameter PGMA_50_-PBzMA_50_ nanoparticles, (b) 79 nm-diameter PGMA_50_-PBzMA_300_ nanoparticles, and (c) 214 nm-diameter PGMA_65_-PBzMA_1000_ nanoparticles.

Inspecting the SEM images shown in Figure 5, it is clear that the evaporation rate (as governed by the RH) is not the only parameter that affects the degree of buckling observed for the final microparticles. More specifically, the mean nanoparticle diameter can also influence their morphology. Figure 8 shows higher-resolution SEM images obtained for microparticles dried at 0% RH when using the 32 nm PGMA_50_-PBzMA_50_, 79 nm PGMA_50_-PBzMA_300_, or 214 nm PGMA_65_-PBzMA_1000_ nanoparticles. Employing the smallest nanoparticles led to microparticles with numerous holes and an annular width of 2.2 μm. Increasing the nanoparticle diameter to 79 nm resulted in dimpled microparticles with annular widths of 4.4 μm. In contrast, smooth dense spherical microparticles were obtained when using the largest nanoparticles. Clearly, the final dried microparticles are much less prone to buckling at a given evaporation rate when employing larger nanoparticles. This trend has also been observed when drying aerosol droplets containing either silica or polystyrene particles.^20^

The *Pe* of an evaporating droplet depends on the size of the included nanoparticles. Larger nanoparticles should have a slower rate of diffusional mixing and therefore increase the degree of surface enrichment during droplet drying.^14^ This is illustrated in Figure 7, where the *Pe* at each RH increases with the nanoparticle diameter. This relationship means that using larger nanoparticles within an evaporating droplet will produce a thicker initial particle shell. The use of larger included nanoparticles will therefore increase both sides of the *a/R* ratio, which determines the degree of buckling. However, using larger nanoparticles also decreases the degree of buckling (see Figure 8). Thus, if the nanoparticle size is increased, the increase in *R* owing to the higher *Pe* is relatively small compared to the change in *a*.

**Figure 9:**
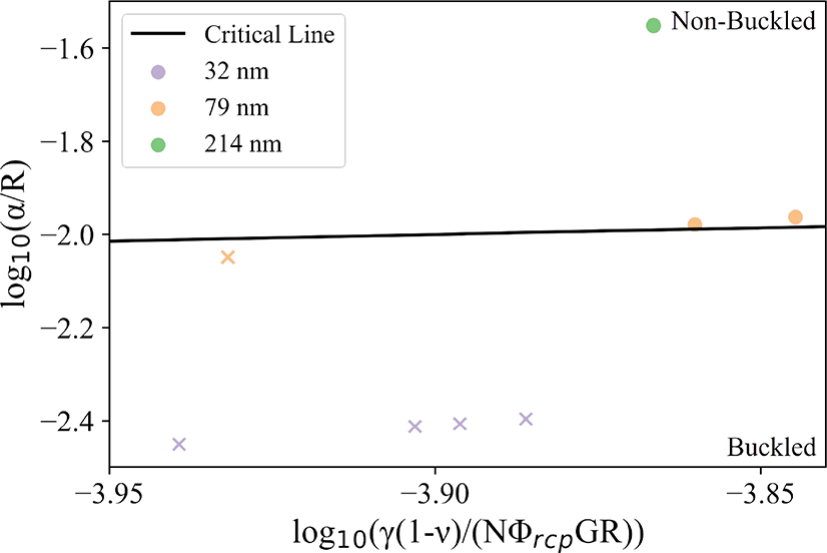
Determination of the buckled and non-buckled regimes for microparticles obtained from drying aqueous aerosol droplets containing PGMA_*x*_-PBzMA_*y*_ nanoparticles. Circular symbols indicate non-buckled microparticles, whereas cross symbols correspond to buckled microparticles. Equation 1 is represented by the “critical line” and the numerical coefficient (*A*) was obtained from fitting. For all cases, ϕ_rcp_ = 0.64, *N* = 6,^45^ γ = 0.072 N m^–1^,^20^ ν = 0.5, and *G* = 18.1 MPa. The values for ν and *G* were obtained from the Young’s modulus and bulk modulus of PBzMA.^46,47^ Microparticle sizes are obtained from FDC data.

To rationalize the impact of the nanoparticle size on the microparticle morphology, Figure 9 examines the relationship between α/*R* and γ(1 – ν)/(*N*ϕ_rcp_*GR*) to compare theoretical estimations of the onset of buckling with experimental data. This analysis assumes that the Darcy pressure drop (Δ*p*_d_) across the particle shell is negligible compared to the maximum capillary pressure (*p*_max_)^20^
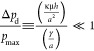
4where κ is the evaporation rate, μ is the liquid viscosity, *h* is the shell thickness, α is the nanoparticle radius, and γ is the surface tension of the droplet. For the systems investigated here, the maximum ratio of the Darcy and capillary pressures is 2.15 × 10^–2^, so the underlying assumption appears to be valid (Table 2).

**Table 2:** Summary of the Darcy/Capillary Pressure Ratios Obtained for Selected Diblock Copolymer Nanoparticles at Various RH Values

nanoparticle type	RH (%)	Darcy/capillary pressure ratio
32 nm PGMA_50_-PBzMA_50_	0	2.15 × 10^–2^
32 nm PGMA_50_-PBzMA_50_	45	1.05 × 10^–2^
79 nm PGMA_50_-PBzMA_300_	0	2.86 × 10^–3^
79 nm PGMA_50_-PBzMA_300_	45	1.40 × 10^–3^
214 nm PGMA_65_-PBzMA_1000_	0	1.44 × 10^–4^
214 nm PGMA_65_-PBzMA_1000_	45	7.03 × 10^–5^

Figure 9 indicates that, for aerosol droplets containing PGMA_*x*_-PBzMA_*y*_ nanoparticles, a reduction in the drying droplet radius at which the shell first forms (owing to the lower *Pe*) and an increase in the nanoparticle size within the droplet both lead to a lower degree of buckling. Indeed, the final observed microparticle morphologies are consistent with our expectations according to eq 4. It is also clear from this plot that the impact of the nanoparticle size on the degree of buckling is much larger for aqueous aerosol droplets containing PGMA_*x*_-PBzMA_*y*_ nanoparticles than the radius shell when it first forms. This supports the qualitative trends observed in Figures 6 and 7 and explains the large difference in the buckling behavior observed for the PGMA_50_-PBzMA_50_ and PGMA_50_-PBzMA_300_ nanoparticles as well as the relatively small difference in the buckling behavior found for PGMA_50_-PBzMA_300_ nanoparticles at 0% and 45% RH.

## 4 Conclusions

This study examines the effect of varying the mean nanoparticle diameter, the nature of the nanoparticle core, and the RH on the drying kinetics of nanoparticle-laden aqueous aerosol droplets and the final morphology of the dried microparticles. The nanoparticle size and chemical composition had no discernible effect on the evaporation rate of such droplets, but the RH influenced the droplet drying kinetics in line with previous studies. Relating the evaporation profiles to SEM images of dried microparticles enabled the elucidation of the relationship between the RH, droplet evaporation rate, and microparticle morphology. Furthermore, the relationship between nanoparticle dimensions and microparticle morphologies was investigated and validated using a model developed by Tirumkudulu.^23^ In summary, the microstructure form of diblock copolymer nanoparticles can be predicted from their size using kinetic data obtained from experiments on single droplets.

## Supporting Information Available

GPC curves for the various diblock copolymer nanoparticles examined in this study, schematics of the EDB and FDC equipment used in this study, and libraries of SEM images used to determine the annular widths and diameters of several types of microparticles (PDF)
