# Supplementary material for: Effect of the Addition of Diblock Copolymer Nanoparticles on the Evaporation Kinetics and Final Particle Morphology for Drying Aqueous Aerosol Droplets
Source: Langmuir. 2023 Dec 21;40(1):734–43. doi: 10.1021/acs.langmuir.3c02930 (PMC10786045; doi:10.1021/acs.langmuir.3c02930)

Supporting Information: Effect of Addition of Diblock Copolymer Nanoparticles on the Evaporation Kinetics and Final Particle Morphology for Drying Aqueous Aerosol Droplets

Barnaby E. A. Miles[1], Derek H. H. Chan[2], Spyridon Varlas[2], , Lukesh K. Mahato[1], Justice Archer[1], Rachael E. H. Miles[1], Steven P. Armes*[2] and Jonathan P. Reid*[1]

[1]School of Chemistry, University of Bristol, Bristol, BS8 1TS, UK
[2]Department of Chemistry, University of Sheffield, Sheffield, S3 7HF, UK
*Corresponding Author for this work.
Email: s.p.armes@sheffield.ac.uk
Email: j.p.reid@bristol.ac.uk

Figure S1. DMF GPC curves obtained for the various diblock copolymer nanoparticles examined in this study. These chromatograms have been grouped according to the chemical composition of the nanoparticle core. (a) Nanoparticles with PBzMA cores. (b) Nanoparticles with a PTFEMA core. (c) Nanoparticles with a PMMA core. In each case the GPC curve recorded for the $PGMA_{50}$ precursor is included for reference.

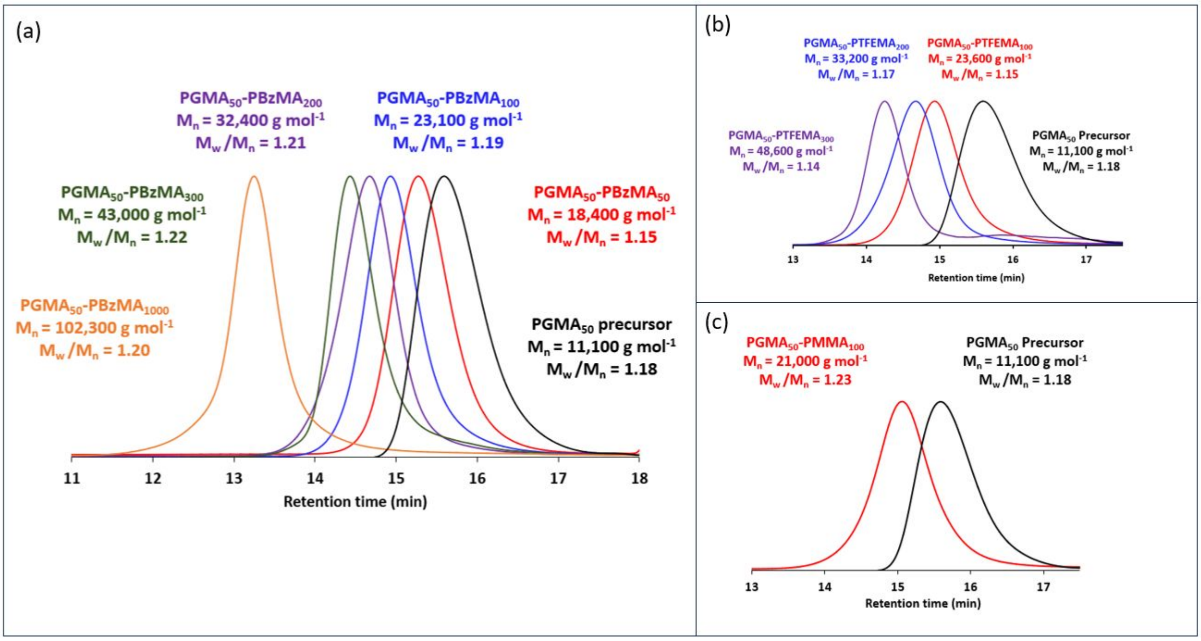

Figure S2. (a) Schematic of a CK-EDB used for measuring droplet drying kinetics. (b) Phase function collected at 0.43 s, indicating a homogeneous spherical droplet. (c) Phase function collected at 5.3 s, indicating a non-spherical, dried particle.

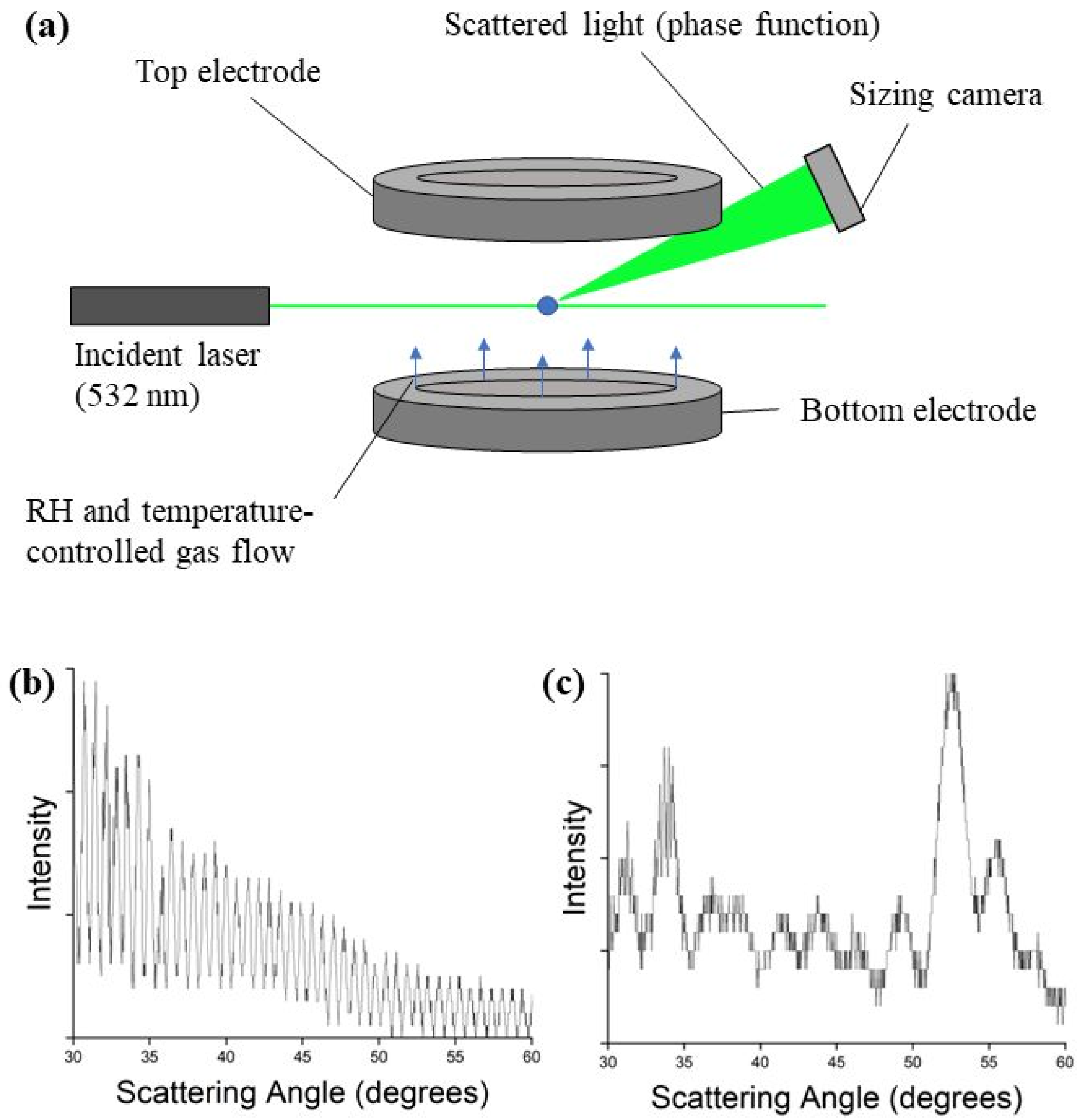

Figure S3. Schematic representation of the FDC set-up used to collect dried microparticles.

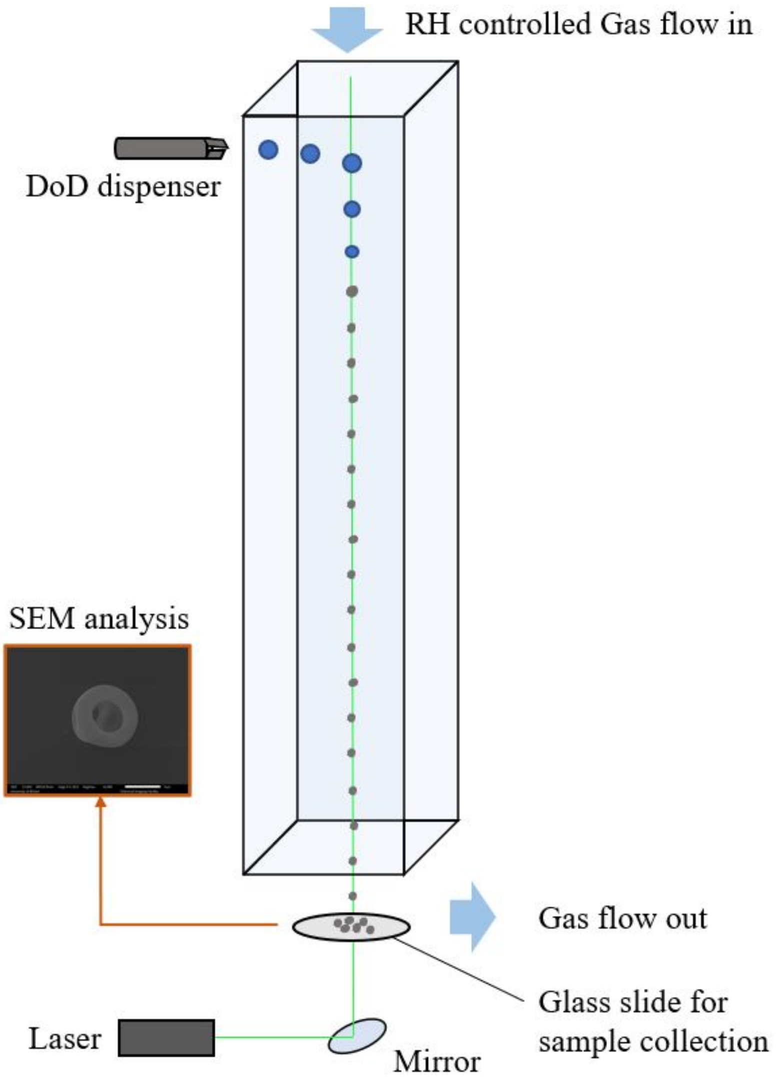

Figure S4. Representative libraries of SEM images of the microparticles obtained from aqueous aerosol droplets (containing 2% w/w nanoparticles) after drying at 293 K. The annular width and particle size can be estimated from such images. (a) For $PGMA_{50}$-$PBzMA_{50}$ nanoparticles dried at 45% RH, the annular width is 4.0 μm (averaged over 6 microparticles). (b) For $PGMA_{65}$-$PBzMA_{1000}$ nanoparticles dried at 0% RH, the mean particle diameter is 7.6 μm (averaged over 20 microparticles). (c) For $PGMA_{65}$-$PBzMA_{1000}$ nanoparticles dried at 45% RH, the mean particle diameter is 7.8 μm (averaged over 10 microparticles).

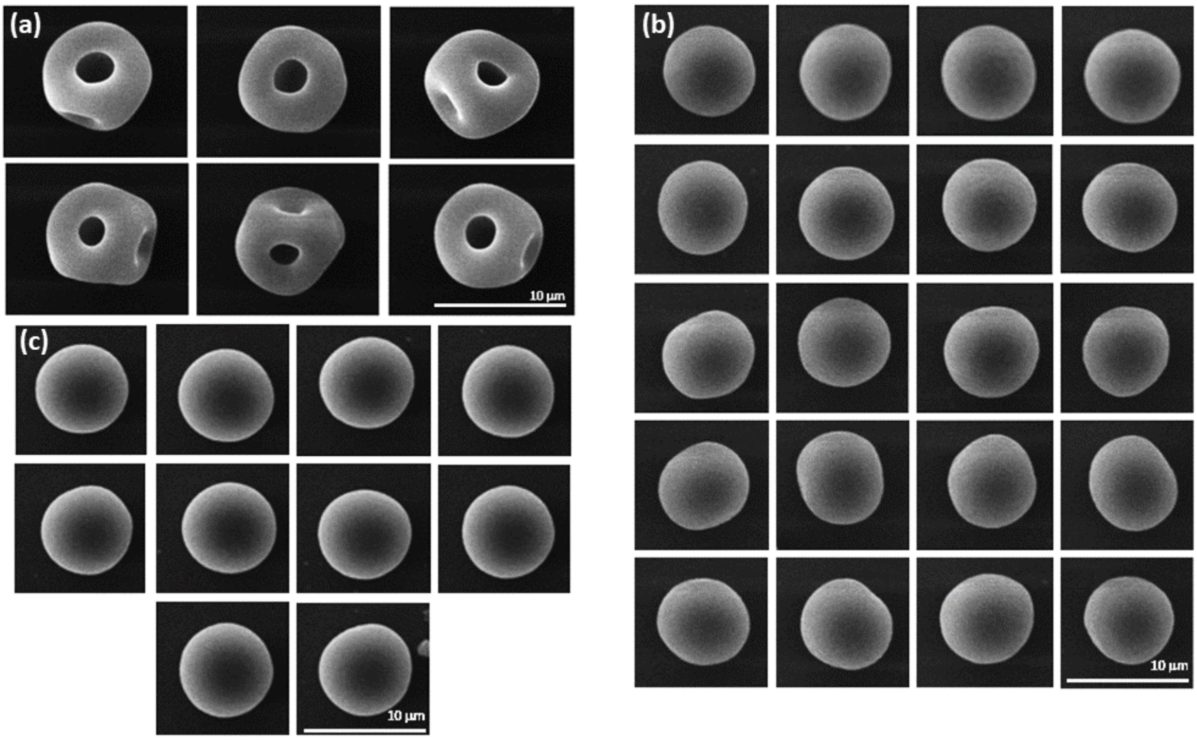

Supplement: Supplementary file 1 — la3c02930_si_001.pdf [file la3c02930_si_001.pdf]
